# Evaluating the immunogenicity and safety of a BiondVax-developed universal influenza vaccine (Multimeric-001) either as a standalone vaccine or as a primer to H5N1 influenza vaccine: Phase IIb study protocol

**DOI:** 10.1097/MD.0000000000006339

**Published:** 2017-03-24

**Authors:** Eva van Doorn, Heng Liu, Tamar Ben-Yedidia, Shimon Hassin, Ildiko Visontai, Stephen Norley, Henderik W. Frijlink, Eelko Hak

**Affiliations:** a Unit of Pharmacotherapy, -Epidemiology and -Economics, University of Groningen, Groningen, The Netherlands; b BiondVax Pharmaceuticals Ltd, Ness Ziona, Israel; c Division of Virology, National Center for Epidemiology, Budapest, Hungary; d Robert Koch Institute, Berlin, Germany; e Department of Pharmaceutical Technology and Biopharmacy, University of Groningen, Groningen, The Netherlands.

**Keywords:** clinical trial, CMI, correlates of protection, HAI, influenza, universal, vaccine

## Abstract

**Introduction:**

Influenza is a major respiratory viral infection of humans with high mortality and morbidity rates and profound economic impact. Although influenza vaccines are generally updated yearly to match the viruses expected in the coming season, genetic mutation and reassortment can result in unexpected novel strains. Therefore, it is important to develop universal vaccines inducing protective immunity to such strains before they appear. This clinical trial is designed to evaluate the safety and immunogenicity of Multimeric-001 (M-001), which contains conserved epitopes of influenza A and B. M-001 is able to induce both humoral and cellular immunity and provides broad strain coverage.

**Methods:**

In a multicenter, randomized, double-blind, and controlled phase IIb trial, 222 healthy volunteers aged 18 to 60 years will be randomized into 3 groups (1:1:1) to receive either 2 intramuscular injections of 0.5 mg M-001 (arm 1), 1.0 mg M-001 (arm 2), or saline (arm 3—placebo), before receiving an investigational (whole virus, inactivated, aluminum phosphate gel [AlPO_4_]-adjuvanted) prepandemic influenza vaccine (H5N1). Primary outcomes are safety and cellular immune responses (cell-mediated immunity [CMI]) induced by M-001, evaluated by multiparametric flow cytometry of intracellular cytokines. The secondary outcome is the serum hemagglutination inhibition (HAI) titer toward the H5N1 vaccine strain. Additionally, exploratory outcomes include evaluation of CMI by quantitative reverse transcription polymerase chain reaction of cytokine mRNA, HAI titers toward H5-drifted strains, serum single radial hemolysis titers toward the H5N1 study vaccine, and the association between CMI markers and antibody response.

**Discussion:**

There is a need for influenza vaccines that give the population a broader protection against multiple strains of influenza virus. M-001 might be such vaccine which will be tested in this current trial as a standalone vaccine and as a pandemic primer. Both cellular and humoral immune responses will be evaluated.

**Trial registration:**

EudraCT number: 2015-001979-46.

## 1 Introduction

Influenza is a globally important respiratory viral infection of humans that is easily transmitted from person to person.^[1]^ As well as causing annual epidemics during the winter period, newly emerging variants can result in global pandemics.^[1,2]^ Infection of humans with influenza virus is characterized by symptoms such as a sudden onset of high fever and coughing. Although most symptoms are self-limiting and resolved within 1 week, influenza can cause severe illness such as pneumonia and otitis media due to the primary influenza infection or to a secondary bacterial infection.^[1,3–6]^ Severe illness can result in hospitalizations and deaths, in particular among individuals who are at high risk for complications (e.g., children younger than 2 years of age, adults aged ≥65 years, pregnant women, and patients with a chronic disease or a weakened immune system).^[2,4,6]^ According to the World Health Organization (WHO) up to 3 to 5 million cases of severe illness occur worldwide during an annual epidemic, resulting in 250,000 to 500,000 deaths, depending on the severity of the influenza season.^[6,7]^ In addition to the morbidity and mortality, these annual epidemics have an enormous economic impact, both from the costs of treatment (direct costs) and the high levels of work absenteeism (indirect costs).^[6,7]^ The total estimated cost of an influenza epidemic in industrialized countries may reach 56.7 million euros per million of population.^[7]^

The most (cost)-effective way to prevent influenza virus infection and severe illness is vaccination.^[6,8]^ Vaccines are designed to induce the immune responses that would be normally induced by natural infection, but without causing the disease.^[9]^ Commercially available influenza vaccines contain 2 viral surface antigens, the hemagglutinin (HA) and neuraminidase (NA).^[8,10]^ HA is responsible for both the attachment of the virus to the sialic-acid-containing receptors on the host cell surface and the entry of the virus into the host cells, whereas NA releases newly formed virus particles from the cell surface.^[8,10]^ The vaccines elicit antibodies toward these proteins, thereby limiting or eliminating their function.^[10]^ Many countries recommend yearly vaccination against seasonal flu for individuals who are at high risk for complications and for people who live with or care for high-risk individuals.^[1,6]^ Each year, the WHO recommends which virus strains should be included in the seasonal influenza vaccine, usually 2 circulating strains of the influenza A (H1N1 and H3N2) virus and 1 or 2 strains of influenza B virus (Yamagata and/or Victoria lineage).^[1,2,6,8]^ The seasonal vaccine must be updated every year due to the minor amino acid changes (antigenic drifts) that occur in the HA and NA viral surface proteins.^[1,8,11]^ These antigenic drifts, resulting from genetic mutations during viral replication, occur gradually over time, eventually result in the unpredictable appearance of new virus strains that may not be effectively recognized by the immune system.^[11]^ Occasionally, an abrupt and major change (antigenic shift) in the influenza A virus may occur resulting in the introduction of a completely new influenza A subtype that has not previously circulated among humans.^[1,2,8,11]^ These viruses are introduced either by the direct transfer of an avian influenza virus to humans or by reassortment between human and avian viruses after coinfection of another animal serving as a “mixing vessel” (e.g., ducks and pigs).^[1,12]^ Being entirely novel, such shifted viruses are poorly recognized (if at all) by the pre-existing immunity to other virus and may be highly contagious and highly pathogenic, resulting in a pandemic characterized by many more cases of severe illness.^[1,2,11]^

One of the most important factors, which influences the type and subtype-specific effectiveness of a vaccine, is the degree of similarity between the vaccine virus strains and the circulating strains.^[13]^ A vaccine can reduce the risk of illness by 50% to 60% among the overall population during seasons when most circulating virus strains are similar to the vaccine strains.^[13,14]^ However, the Centers for Diseases Control and Prevention (CDC) in the United States estimated that the vaccine effectiveness for the Northern hemisphere in the 2014 to 2015 influenza season was only 23% (95% confidence interval [CI]: 14–31) in the general population.^[15]^ This low efficacy is considered to be the result of the mismatch between the H3N2 contained in the vaccine (A/Texas/50/12) and the circulating H3N2 virus (A/Switzerland/9715293/13) and resulted in the highest recorded rate of flu-associated hospitalization (266.1 per 100,000) among adults aged 65 and older in the United States since the CDC started tracking data.^[13,15–18]^ This mismatch highlights the need for influenza vaccines that give the population a broader protection against multiple strains of influenza virus: a universal vaccine.^[10,19]^

Multimeric-001 (M-001), developed by BiondVax Pharmaceuticals Ltd. (Israel), is designed to be a universal influenza vaccine that confers immunity and protection against a broad range of influenza viruses. M-001 is a single recombinant protein containing 9 B- and T-cell conserved epitopes (epitopes that do not undergo antigenic change) from the HA, nucleoprotein, and matrix 1 proteins of both the influenza A and B virus strains. The vaccine induces both humoral and cellular immunity.^[20,21]^ In this phase IIb trial, the immunogenicity and safety of M-001 and its administration prior to an aluminum phosphate gel (AlPO_4_)-adjuvanted H5N1 investigational influenza vaccine product (Fluart Innovative Vaccines Ltd., Hungary) will be evaluated in healthy adults (aged 18–60 years). Fluart H5N1 vaccine is highly immunogenic at doses of 6 and 12 μg and hence, a suboptimal dose of the vaccine (3 μg) will be used to evaluate the dose sparing potential of M-001 and to show its ability to enhance immunogenicity of the strain-specific vaccine.^[22]^ The immunogenicity of M-001 both as a standalone vaccine and as a pandemic primer will be evaluated. In addition, the association between the cell-mediated immunity (CMI) markers and the antibody responses will be evaluated as an exploratory endpoint. The safety will be evaluated based on the reported adverse events (AEs) and serious adverse events (SAEs) throughout the whole study period.

## 2 Method/design

### 2.1 Study design

**Figure 1:**
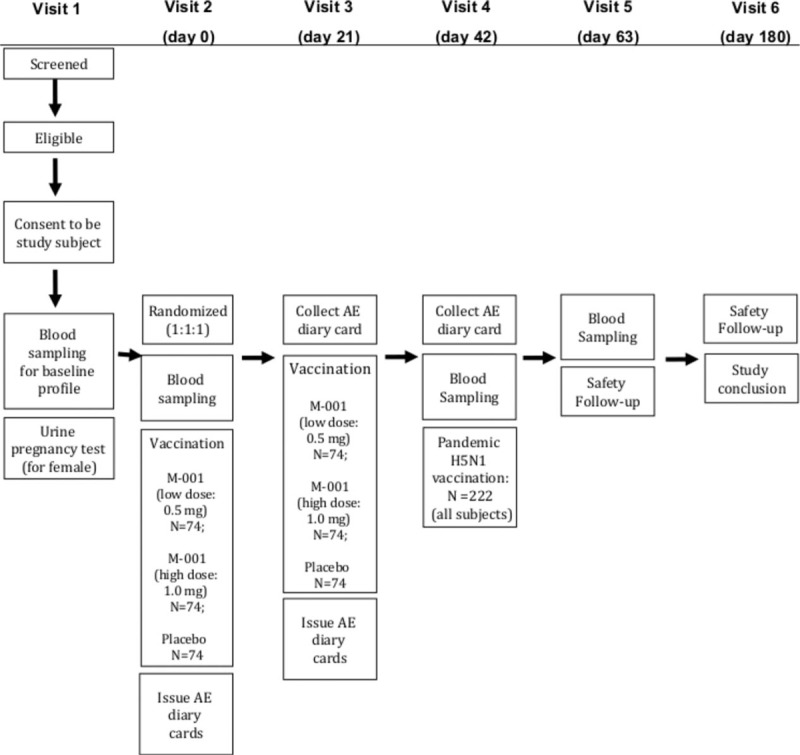
Flow chart describing the study's design.

The study is a multicenter, randomized, double-blind, and controlled phase IIb trial involving 222 healthy volunteers aged 18 to 60 years over a period of 187 days (from screening to study conclusion). Individuals volunteering to participate in the study will be screened before enrollment in the trial. Subject eligibility will be assessed during the screening and written consent will be obtained (Fig. 1). The eligible subjects will be randomized to receive 2 administrations of M-001 at low or high dose, or saline as placebo. All participants will receive an investigational AlPO_4_-adjuvanted, inactivated whole virus prepandemic H5N1 vaccine as a third administration to evaluate whether M-001 has a priming effect. The H5N1 vaccine is based on a clade 1 virus A/Vietnam/1194/04. Each administration will be given intramuscularly (i.m.) 21 ± 2 days apart. Blood samples will be taken from all subjects on days 0 (before the first vaccination), 42 (21 days after the second vaccination), and 63 (21 days after the H5N1 vaccination) for serum and peripheral blood mononuclear cells (PBMCs) to evaluate the influenza-specific cellular and humoral immune responses. Additionally, questionnaires/diaries concerning AEs will be issued on days 0 and 21 to subjects for the collection of solicited (e.g., fever, chills, joint pain, muscle pain, sore throat, fatigue, injection site reactions) and unsolicited AEs. The cards will need to be completed by the subjects and returned on day 21 (second vaccination of study vaccine) and day 42 (H5N1 vaccination). Unsolicited AEs and SAEs will be followed throughout the whole study period and thereby AEs from both the study vaccine and H5N1 vaccine. The study will be conducted at the St. Istvan and St. Laszlo Hospital of Budapest (Hungary) and another Hungarian satellite trial site (Béke Hospital, Budapest).

### 2.2 Participants

Individuals will be recruited using advertising material approved by the ethics committee. Healthy males and females between the ages of 18 and 60 are eligible for study participation (Table 1). Pregnant or breast-feeding women are excluded. Other exclusion criteria include individuals who have received an influenza vaccine or have experienced influenza-like illness within the 6 months prior to the study, those who are receiving medicines or treatments that may affect the evaluation of their immune responses or those who have a history of chronic disease and/or immune system disorder (Table 1). Women of childbearing potential and men must agree to practice adequate contraception throughout the study treatment and for at least up to days 51 and 111 of the trial, respectively, since no reproduction toxicity data are known yet. Furthermore, individuals should be able to understand and comply with the study procedures. In addition, individuals will only be included if the individual provides a signed informed consent form after receiving a detailed explanation of the study protocol prior to any study procedures. In case of uncertainty about the medical status of an individual regarding any of the exclusion criteria mentioned, the primary care physician will be consulted. Consultation of the primary care physician is included in the consent form and only concerns medical information about the exclusion criteria.

**Table 1:**
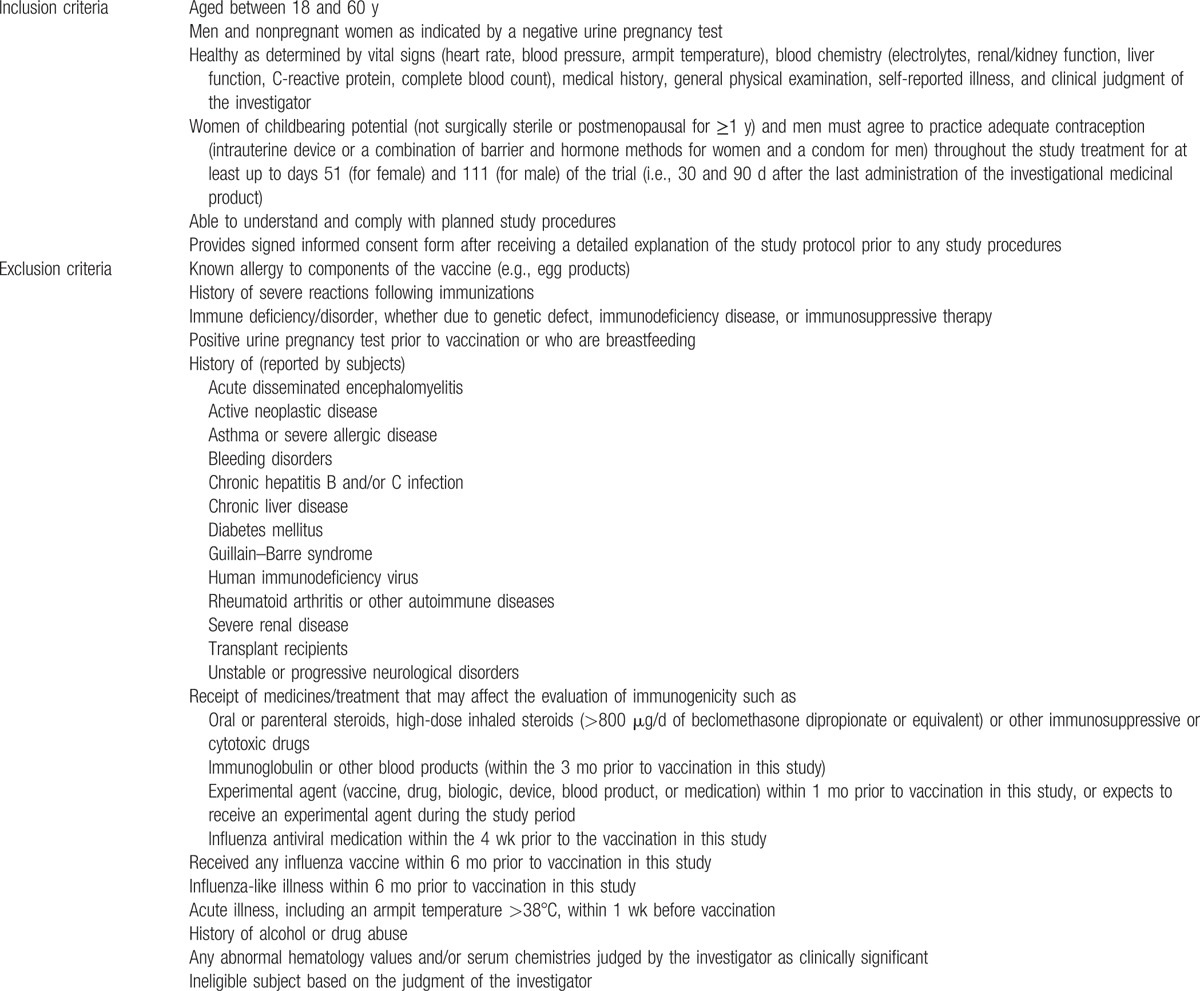
Inclusion and exclusion criteria of individuals.

### 2.3 Screening and baseline assessment

Baseline assessment during the screening visit will include demographics (i.e., age, gender, and ethnicity), general physical examination, self-reported medical and medication history, history of influenza vaccination, and alcohol, drug, and cigarette consumption. This is to ensure that subjects enter the trial in a healthy condition. A blood sample will be taken at the screening visit for laboratory tests including hematology (e.g., hemoglobin, hematocrit, total and differential leukocyte count, red blood cell count, platelet count) and serum chemistry (e.g., uric acid, creatinine, cholesterol, triglycerides, sodium, potassium). Subjects having an abnormal result for any of the tests, upon consultation with the principal investigator, would be excluded from the study. In addition to the blood sampling, females will undergo a urine pregnancy test. Each screened subject will receive a sequential, unique 5-digit screening number which they will retain, whether or not they are ultimately randomized to receive treatment. Serum samples from the collected blood at visits 2 and 5 (days 0 and 63) will be stored until the end of the trial when serology will be performed to detect antibodies to the H5N1 virus and against the 2015–2016 circulating influenza virus strains in order to identify subjects having had an asymptomatic influenza infection prior to the start of the trial. On day 180 of the trial, each participant will receive a phone call for safety follow-up and then the study will terminate.

### 2.4 Interventions

Individuals who meet the eligibility criteria will be randomized (1:1:1) to receive either 0.5 mg M-001 (low dose), 1.0 mg M-001 (high dose), or saline (placebo control). All subjects will receive 2 i.m. administrations of the test vaccine, or saline, followed by 1 i.m. administration of an investigational (whole virus, inactivated, AlPO_4_-adjuvanted) prepandemic influenza vaccine (H5N1) produced by Fluart Innovative Vaccines Ltd (formerly Omninvest Ltd). The prepandemic vaccine contains 3 μg HA, which is half of the dose of the pandemic vaccine licensed in Hungary.

### 2.5 Outcomes

#### 2.5.1 Primary outcomes

The trial has 2 primary outcomes. The first primary outcome is the cellular immune response elicited by M-001, evaluated by multiparametric flow cytometry analysis in all subjects on days 0 and 42. Multiparametric flow cytometry will be performed after 24 h in vitro stimulation of PBMC with vaccine antigen. Markers including clusters of differentiation 3 (CD-3), CD-4, and CD-8 will be used to define cell lineage, and interferon gamma (IFN-γ), interleukin (IL)-2, IL-4, and tumor necrosis factor alpha (TNFα) will be used to detect specific cytokine responses. The other primary outcome is safety evaluation. This includes the solicited AEs in all subjects until 21 days after the last dosing of the study vaccine and the unsolicited AEs and SAEs in all subjects for 180 days after the first injection. The investigator will assess the intensity of the AE and the relationship between the tested vaccine and the occurrence of the AE (Table 2).

**Table 2:**
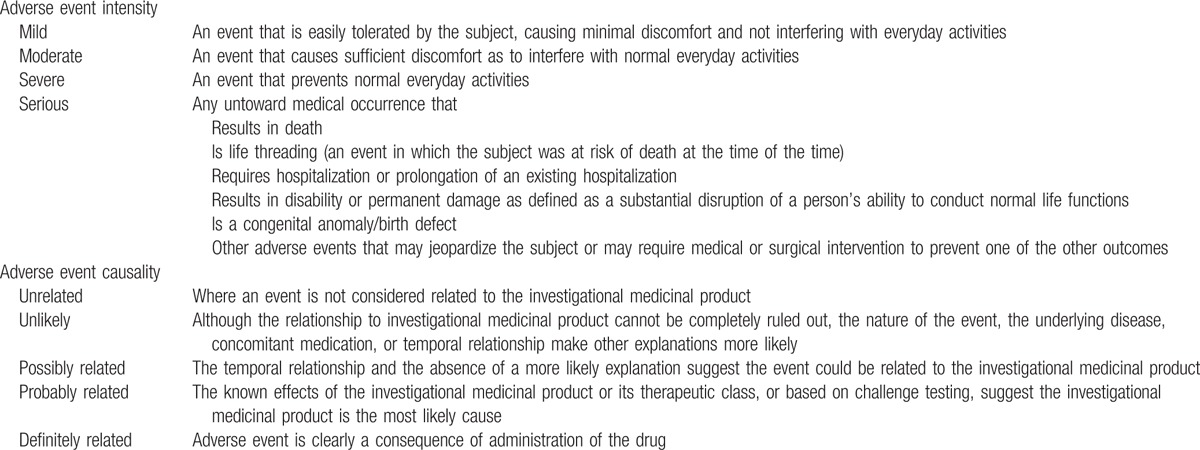
Adverse events’ classification.

#### 2.5.2 Secondary outcomes

The secondary outcome is serum hemagglutination inhibition (HAI) titers specific for the H5N1 study vaccine strain (A/Vietnam/1194/2004) in all subjects on days 0 and 63 (21 days following the H5N1 vaccination). HAI tests will be performed by standard procedures with chicken red blood cells and 4 HA units of virus/well.^[23]^

#### 2.5.3 Exploratory outcomes

The following exploratory outcomes are considered; (1) cellular immune response elicited by M-001, with or without prepandemic H5N1 vaccine, evaluated by quantitative reverse transcription polymerase chain reaction (qRT-PCR) of cytokine mRNA on days 0, 42, and 63 in a subset of 60 subjects; (2) serum HAI titers toward H5 drifted strains evaluated on days 0 and 63 in all subjects; (3) serum single radial hemolysis (SRH) titers evaluated on days 0 and 63 in all subjects toward the H5N1 vaccine strain; and the association between the CMI markers analyzed and (4) the HAI responses.

### 2.6 Sample size calculation

Though a variety of immune parameters will be analyzed during the trial, the minimum sample size is determined on the basis of the expression of IFN-γ producing CD-8+ cells, as this T-cell subset is important for influenza viral clearance.^[24,25]^ We estimated a background rate of 5% in the placebo group and it is expected that the M-001 vaccine will result in at least 5 times higher rates, or an absolute rate of 25% responders in the vaccinated arms.^[21]^ To be able to detect this with a power of 80%, approximately 60 subjects per arm are needed based on a 2-group continuity corrected test with a 0.05 two-sided significance level. Taking 15% loss of follow-up into account, a total of 222 subjects (74 subjects per treatment group) is required. It should be noted that estimates are based on data analysis from a previous trial where multiparametric flow cytometry analysis was performed on only a small sample size of 10 elderly participants.

### 2.7 Randomization

Eligible subjects will be randomized 1:1:1 to 1 of the 3 treatment groups. Within each treatment group block randomization will be done for participants aged <50 years and ≥50 years to achieve an approximately balanced age distribution.^[26]^ Randomized subjects will be allocated the next sequential 3-digit randomization number available at the trial site which is identical to the treatment number allocated by the randomization list.

### 2.8 Blinding

The study will be double-blinded. To maintain blinding, the preparation of the M-001 formulations and control will be performed by an unblinded qualified person (QP) other than the person giving the injection. The reason for this is that M-001 and saline differ in appearance. The unblinded QP will prepare the dosing solutions and will use the study randomization code to assign each subject to the appropriate treatment group. The unblinded QP will not reveal the treatment code to other study personnel or perform any other study treatment-related activities. The unblinded QP will prepare syringes containing the study medication assigned by the randomization scheme. The syringe content will be hidden by a label, which includes the subject's randomization number, initials, and date of birth. An identical label will be attached to the top of the case report form. This material will be given to the blinded study personnel administering the injections. The pharmacy file, including the randomization list, vaccine supplies, and all associated documentation, will be stored in a locked cabinet within the pharmacy to which only the unblinded pharmacy personnel have access.

### 2.9 Statistical analysis and report

A statistical analysis plan (SAP) will be developed according to the Consolidated Standards of Reporting Trials 2010 guidelines before the database lock.^[27]^ Both intention-to-treat (ITT) and perprotocol analysis will be performed for the analyses of reported AEs and SAEs, the measured serum HAI titers, SRH titers, and the cellular immune response measured by both multiparametric flow cytometry and qRT-PCR assays. ITT analysis will include all vaccinated subjects who receive the first vaccination. Perprotocol analysis will include the subjects who complete the assigned schedule of vaccination, who meet the inclusion and exclusion criteria, and who comply with the procedures defined in the protocol (i.e., vaccinated according to their randomized assignment, not vaccinated with any vaccine or treated with any medication not foreseen or forbidden in the protocol, free of underlying medical conditions forbidden by the protocol) until the days of assessment and have not broken the randomization code. Both analyses will be conducted to assess differences from baseline with their corresponding 95% CIs. Differences in continuous variables will be tested using the analysis of variance (ANOVA)/T-tests or distribution-free tests. In addition, subgroup analyses are preplanned according to age (<50 and ≥50 years) and ANOVA or Kruskal–Wallis test will be applied to test for potential differences between the treatment groups. All calculations will be performed using SAS for Windows. The measured variables and derived parameters will be listed individually by subject number and treatment group. The data will be summarized in appropriate tables presenting sample size, arithmetic mean, standard deviation, minimum, median, and maximum values by treatment group for continuous data and absolute and relative frequency by treatment group for categorical data.

### 2.10 Data management

An interactive website will be established which will be the major communication instrument ensuring the integration of program communication and the trial study. In addition, electronic case report forms will be developed in accordance with the good clinical practice (GCP) standards for electronic data entry and flexible data export, and will be integrated into the interactive website. The data entered will be backed up on a daily basis, be subject to secure access control management to allow secure entry, access, analysis, and export of data by users regardless of their locations and be subject to plausibility and consistency checks during the entry process to enforce high data integrity. All nominal subject data will be anonymized to ensure personal data protection. If several reports taken at different times have to be correlated, pseudo-anonymization will be used.

### 2.11 Ethics and disseminations

Ethical approval and authorization of the trial is obtained from the Clinical Pharmacology Ethics Committee (Klinikai Farmakológiai Etikai Bizottság) and the Hungarian National Institute of Pharmacy and Nutrition (Országos Gyógyszerészeti és Élelmezés-egészségügyi Intézet), respectively. The Institutional Ethics Committee of St. Istvan and St. Laszlo Hospital was also informed.^[28]^ The trial will be performed in compliance with GCP, the Declaration of Helsinki and relevant Hungarian national laws. Written informed consent will be obtained from subjects before participation in the trial. The original subject signed and dated informed consent form will be stored by the site for 10 years. Other trial data will also be stored for 10 years. The trial results will be made suitable for publication in international scientific journals.

## 3 Discussion

The main objective of this phase IIb trial is to evaluate the safety and immunogenicity of M-001 as a standalone universal vaccine and when used as a primer for a pandemic influenza vaccine. Currently, the standard criterion to determine influenza vaccine efficacy is based on HAI titer.^[25,29,30]^ However, this criterion is not applicable for M-001 since only conserved epitopes without hemagglutination and neutralization capacity are included in it.^[20,25]^ Previous trials using M-001 have shown that it induces influenza-specific cellular immune responses, which are suggested to be important for protection against influenza disease.^[31]^ The vaccine efficacy of M-001 will therefore be evaluated by multiparametric flow cytometry analysis in the current trial. Flow cytometry is chosen because the assay provides a sensitive technique for qualitative and quantitative analyses of cellular immune responses.^[25,32]^ Only 1 blood sample is needed to simultaneously measure CD-3, CD-4, and CD-8 T-cells and the functional markers IFN-γ, IL-2, IL-4, and TNF-α for both CD-4 and CD-8 T-cells responses.^[24,25,33]^ The analysis will be standardized and validated before analyzing the clinical samples. In addition to flow cytometry, detection of IFN-γ and granzyme B mRNA expression by qRT-PCR assay will be performed. This method has recently been shown to be a highly sensitive measure for the detection of CMI in guinea pigs immunized intradermally with heat-inactivated varicella-zoster virus.^[34]^ The ultimate aim is to develop a set of correlates of protection that could improve the translation of immunological data from small clinical trials in humans into conclusions about protective efficacy. Currently large clinical trials are required for assessing vaccine efficacy (reduction of illness), since the incidence of influenza is only ∼5%. If such immunological correlates of protection could be identified, they could serve as a surrogate measure of protection in comparatively small trials.

M-001 has been tested as a primer for a seasonal trivalent inactivated influenza vaccine (TIV) in adults aged 55 to 75 years and the elderly (over 65 years of age).^[21]^ These studies showed that TIV, which was administrated 3 weeks after receiving 1 or 2 administrations of M-001 or placebo, with an interval of 21 ± 2 days, induced higher HAI antibody titers in individuals primed with M-001. In addition, M-001 also increased the TIV seroconversion rate (percentage of participants with a minimum 4-fold increase in the HAI titer and ≥1:40 HAI antibody titers) and broadens the antibody responses toward influenza strains other than the TIV strains. This indicates that M-001 has the potential to act as a primer for influenza vaccines in general which is especially important in the case of weakly immunogenic HA-based influenza vaccines, such as a pandemic vaccine. Since it is not feasible to determine the clinical efficacy of a pandemic vaccine in a clinical trial and it takes several months to manufacture a pandemic vaccine, a prepandemic primer such as M-001 represents a breakthrough in pandemic preparedness.^[29]^ Stockpiled M-001 can be administered immediately upon pandemic alert to prime the immune response for the pandemic vaccine still under production, irrespective of the identified pandemic strain which will be included in the pandemic vaccine. The success of this pandemic prime-boost approach is expected to greatly reduce the illness and economic loss caused by an upcoming pandemic. To assess the efficacy of this prime-boost approach, HAI titers toward the H5N1 study vaccine strain (secondary outcome) and other H5 drifted strains (exploratory outcome) induced by the investigational H5 pandemic vaccine, with or without M-001 priming, will be evaluated.

Finally, M-001 is a recombinant product, which is produced in *Escherichia coli* using standard fermentation and purifications methods.^[20]^ This is a simple, scalable manufacturing process. The vaccine production takes only 6 to 8 weeks and it has the possibility of flexible manufacturing year-round, meaning that production can be planned in accordance with market demands, enabling national stockpiles to be maintained for all income class countries.^[20,21,35]^ Furthermore, people with egg-allergies can be vaccinated with the standalone vaccine as the vaccine is not produced in embryonated chicken eggs.^[1]^
